# Longitudinal 4D Flow MRI‐Derived Wall Shear Stress in Patients With an Abdominal Aortic Aneurysm

**DOI:** 10.1002/jmri.70248

**Published:** 2026-02-11

**Authors:** E. Aalbregt, W. Stehling, E. M. Schrauben, J. van Schuppen, L. J. Meijboom, R. A. P. Takx, A. J. Nederveen, P. van Ooij, V. Jongkind, K. K. Yeung

**Affiliations:** ^1^ Department of Surgery, Amsterdam University Medical Center Location University of Amsterdam Amsterdam the Netherlands; ^2^ Department of Radiology and Nuclear Medicine, Amsterdam University Medical Center Location University of Amsterdam Amsterdam the Netherlands; ^3^ Amsterdam Cardiovascular Sciences Amsterdam the Netherlands; ^4^ Department of Radiology and Nuclear Medicine, Amsterdam University Medical Center Location Vrije Universiteit Amsterdam the Netherlands

**Keywords:** 3D cine bSSFP, 4D flow MRI, abdominal aortic aneurysm, longitudinal, wall shear stress

## Abstract

**Background:**

4D flow MRI‐derived parameters such as wall shear stress (WSS) may improve abdominal aortic aneurysm (AAA) progression prediction. Validation of this biomarker is needed, but longitudinal data are lacking.

**Purpose:**

Investigate longitudinal WSS changes and associations with AAA morphology.

**Study Type:**

Prospective.

**Subjects:**

Fifteen patients (mean age 68.7 ± 8.9 years; 1 female) with AAA > 30 mm in diameter.

**Field Strength/Sequence:**

3.0 T, 4D flow MRI, 3D cine balanced steady state free precession, and Dixon.

**Assessment:**

Patients had baseline and follow‐up MRI studies, separated by 6 months. A previously developed automated post‐processing 4D flow MRI software was utilized to assess WSS, total lumen, and thrombus volumes. Maximum diameter in the anteroposterior (AP) and left–right (LR) directions was measured by three radiologists (with 13, 12, and 3 years of experience) based on the Dixon MRI at both time‐points. Statistically significant growth was defined as an increase exceeding twice the standard error of the measurement. Baseline and follow‐up 3D WSS maps were visually compared to identify potential temporal differences.

**Statistical Tests:**

The Wilcoxon signed‐rank test was applied to evaluate differences in diameter, volume, and WSS measurements between baseline and follow‐up. Spearman's rank correlation coefficients were calculated to assess correlations between (half‐year change in) WSS values and AAA diameter and volumes.

**Results:**

Maximum AAA diameter increased significantly from baseline to follow‐up. Also, a significant difference was observed in maximum peak WSS between baseline and follow‐up. A significant inverse correlation was observed between the change in minimum peak WSS and AAA lumen volume over follow‐up. Visual assessment of WSS maps may improve 4D flow–based AAA surveillance by identifying localized changes.

**Data Conclusion:**

Maximum AAA diameter increased over the study period. Changes in WSS were inversely associated with changes in AAA lumen volume over follow‐up but not with maximum AAA diameter. Longer follow‐up is needed to assess the potential of WSS as a biomarker for AAA progression.

**Evidence Level:**

Level 2.

**Technical Efficacy:**

Level 3.

## Plain Language Summary

To prevent the life‐threatening rupture of an enlarged aorta, better risk prediction is needed. Currently, risk prediction is based on the diameter of the enlarged aorta. 4D flow MRI is an imaging technique that provides quantitative information about blood flow and may improve risk assessment. In this study, changes in 4D flow MRI–derived parameters and AAA size measurements were assessed over a half‐year interval. Diameter growth was observed but no correlations with longitudinal changes in 4D flow–derived parameters were observed. Longer follow‐up is required to determine whether 4D flow MRI‐derived parameters could improve surveillance of enlarged aortas.

## 1 Introduction

When the maximum diameter of an abdominal aortic aneurysm (AAA) reaches a certain threshold (5.0 cm for women and 5.5 for men), elective surgery can be considered to prevent rupture [1]. However, up to 10% of patients who experience an AAA rupture may have a maximum aneurysm diameter below the threshold [2]. In addition, only 10% of patients with AAA diameters between 6.0 and 6.9 cm experience rupture within 1 year [3]. This indicates that the maximum diameter threshold might not be specific enough to capture the complex and multifactorial mechanisms of AAA pathophysiology and rupture. Improved rupture risk prediction could prevent unnecessary interventions and their consequent complications [4, 5], and allow focus on those patients at true risk.

Quantitative MRI has the potential to improve rupture risk prediction in AAA by providing new quantitative biomarkers in addition to established morphological parameters. Four‐dimensional (4D) flow MRI enables visualization and quantification of blood flow in three directions over time (i.e., over the cardiac cycle) [6]. Based on measured blood flow velocity, the wall shear stress (WSS) can be estimated. WSS is the frictional tangential force exerted by the flowing blood on the vessel wall, and vice versa, per surface area [7]. WSS plays an important role in maintaining the structural integrity of healthy vessel walls. Endothelial cells lining the arterial wall act as mechanoreceptors [8, 9] and respond to WSS [10, 11], initiating protective pathways to preserve wall integrity. These pathways can be disrupted when WSS is excessively high or low. Regions of low WSS have been associated with aortic aneurysm progression and rupture [12, 13, 14].

WSS is a promising biomarker for AAA progression; however, its validation is limited by the lack of longitudinal studies assessing WSS changes during aneurysm progression in the abdomen, leaving the relationship between disease progression and evolving hemodynamics incompletely understood [15]. In the ascending aorta, several studies have reported assessing aneurysm growth and WSS parameters [16, 17, 18, 19]. However, results from these studies do not translate well to AAA because of different hemodynamics due to the presence of tortuous anatomy, the iliac bifurcation, and intraluminal thrombus [20, 21]. The exact role of thrombus in AAA pathophysiology is controversial [22]. It has been hypothesized that AP differences in WSS might contribute to thrombus formation [22, 23].

The aim of the current explorative study was to assess longitudinal changes in 4D flow MRI‐derived WSS and to investigate associations with maximum AAA diameter, the current clinical reference standard, and other morphological parameters over a 6‐month period.

## 2 Materials and Methods

### 2.1 Study Design and Population

This study received approval from the Amsterdam UMC medical ethics board in accordance with the Dutch Medical Research Involving Human Subjects Act (WMO). All participants provided written informed consent. The study, part of a prospective single‐center investigation (ClinicalTrials.gov ID: NCT0596711), recruited patients with an asymptomatic AAA of at least 30 mm in diameter or < 1.5 times the proximal aorta diameter [1], as determined by CT, ultrasound (US), or MRI examinations performed as part of routine clinical care. Included patients were enrolled between December 2023 and May 2024 and scanned at enrollment (baseline) and 6 months later. Exclusion criteria included severely impaired renal function (eGFR < 20 mL/min/1.73m^2^), supra‐ or pararenal AAA, previous AAA repair, untreated cardiac arrhythmias, inflammatory, infectious, or mycotic AAA, untreated vasculitis, or a connective tissue disorder.

### 2.2 MRI Acquisition

All MRI examinations were conducted using a 3.0 T Philips MR7700 MRI system, featuring software version 5.9 (Philips Medical Systems, Best, The Netherlands), along with a dStream 16‐channel Torso coil and a 12‐channel posterior coil. The participants underwent two abdominal MRI scans that included free‐breathing 4D flow MRI, free‐breathing 3D cine balanced steady state free precession (bSSFP) MRI [24] and a two‐point Dixon MRI with breath hold. The reproducibility of the employed 4D flow technique has been previously shown [25]. As part of the scanning protocol, patients were administered a gadolinium‐based contrast agent (Dotarem, Guerbet) for dynamic contrast‐enhanced MRI (0.2 mL/kg patient weight at a rate of 2 mL/s) followed by a flush of 20 mL saline at the same rate. This administration occurred approximately 15 min prior to the 4D flow MRI acquisition and 25 min before the 3D cine bSSFP MRI acquisition. Dixon MRI was acquired before and approximately 30 min after contrast administration. Electrocardiography (ECG) signals were recorded to facilitate retrospective cardiac binning. If the ECG signal was unstable, a peripheral pulse signal was used as an alternative. Respiratory motion was compensated in 4D flow MRI via gating to end‐expiration with a lung‐liver navigator, while in 3D cine bSSFP, retrospective motion correction was used [26]. The sagittal field of view (FOV) used for 4D flow and 3D cine bSSFP was positioned to cover at least the area from the renal arteries to the aortic bifurcation into the iliac arteries. Acquired and reconstructed spatial resolutions were respectively 1.6 mm^3^ and 1.0 mm^3^ for both sequences. A 2D phase contrast scout scan with a FOV positioned just beneath the renal arteries was acquired to identify potential aliasing at the selected velocity encoding of 52 cm/s. The FOV was adjusted to individual patient anatomy for both 3D cine bSSFP and 4D flow MRI, with partial exclusion of the iliac arteries. With this approach, scan duration was 7–11 min for 4D flow MRI and 4–6 min for 3D cine bSSFP depending on aneurysm size. The two‐point Dixon MRI was acquired before and after contrast injection with a reconstructed spatial resolution of 1 × 1 × 1.75 mm and scan duration of 15 s. For detailed acquisition parameters we refer to Supporting Information Table S1.

### 2.3 MRI Post‐Processing

**FIGURE 1:**
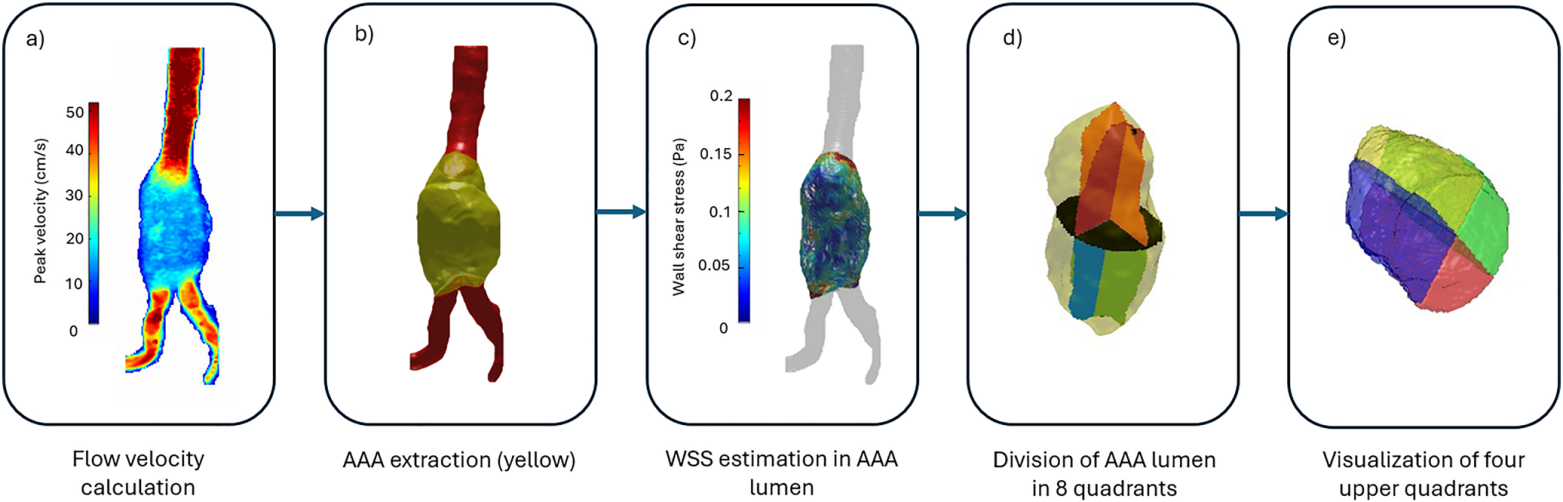
Visualization of several steps within the post‐processing pipeline. (a) calculation of flow velocity at the peak systolic timeframe over the entire segmented volume, (b) extraction of the AAA (in yellow) including lumen + thrombus, (c) calculation of WSS at the peak systolic timeframe within the extracted AAA lumen volume, (d) division of the extracted AAA lumen volume into eight regions, (e) visualization of the four upper quadrants.

The raw 4D flow MRI and 3D cine bSSFP data were exported and reconstructed offline using MATLAB (version R2021a, Mathworks, Natick, MA, USA) alongside ReconFrame 5.4.1 (Gyrotools, Zurich, Switzerland). The image reconstruction involved a non‐linear compressed sensing algorithm [27] implemented in the Berkeley Advanced Reconstruction Toolbox (BART) [28]. A previously developed automated post‐processing pipeline for 4D flow MRI in AAA patients was utilized in the current study (https://github.com/schrau24/AAA_4Dflow_auto) [29]. The aorta, AAA, and thrombus (when present) were segmented from 3D cine bSSFP data using an nnU‐Net‐based method [25, 29, 30]. The resulting segmentations and the 4D flow MRI data served as input for the automated pipeline. The pipeline followed these main steps: cropping of the data to accelerate processing, linear registration of 4D flow MRI to 3D cine bSSFP, unwrapping and denoising of the 4D flow data to respectively remove potential aliasing and reduce noise, centerline extraction based on a thinning operation, aneurysm extraction based on diameter measurements (threshold for AAA was set to 3 cm [1]) in each orthogonal cross‐section along the centerline and lastly calculation of WSS values within the extracted aneurysm, see Figure 1a–c. Mean and peak WSS were calculated within the extracted aneurysm per time frame [25, 31]. To avoid data distortion by outliers, the peak WSS for each time frame was determined using the 95th percentile of the values. From these mean and peak WSS values for each time frame, the frames with the lowest and highest values were identified, resulting in four variables: the minimum mean WSS, the maximum mean WSS, the minimum peak WSS, and the maximum peak WSS. In previous studies, regional differences have been described in the context of complex vascular geometries [16, 32, 33, 34]. Hence, the extracted aneurysm was divided into eight regions by first separating it into upper and lower halves, followed by dividing each half into anterior, posterior, right, and left quadrants, see Figure 1 d‐e. Processing time was approximately 30 min per patient utilizing 6 CPU cores and 45GB of RAM. For further details regarding the post‐processing and 3D cine bSSFP–based nnU‐Net segmentation we refer to a previous study [29].

### 2.4 Diameter and Volume Measurements

Diameter data were collected to assess potential aneurysm growth. Three radiologists (JvS, LM, and RT with respectively 13, 12, and 3 years of experience as certified cardiovascular radiologists) each measured the maximum diameter of the AAA both in the anteroposterior (AP) and left–right (LR) direction based on the post‐contrast Dixon MRI. Measurements were done on a Sectra Workstation (version 25.2; Linköping, Sweden). The final values used in analyses were obtained by averaging the measurements of the three observers.

**FIGURE 2:**
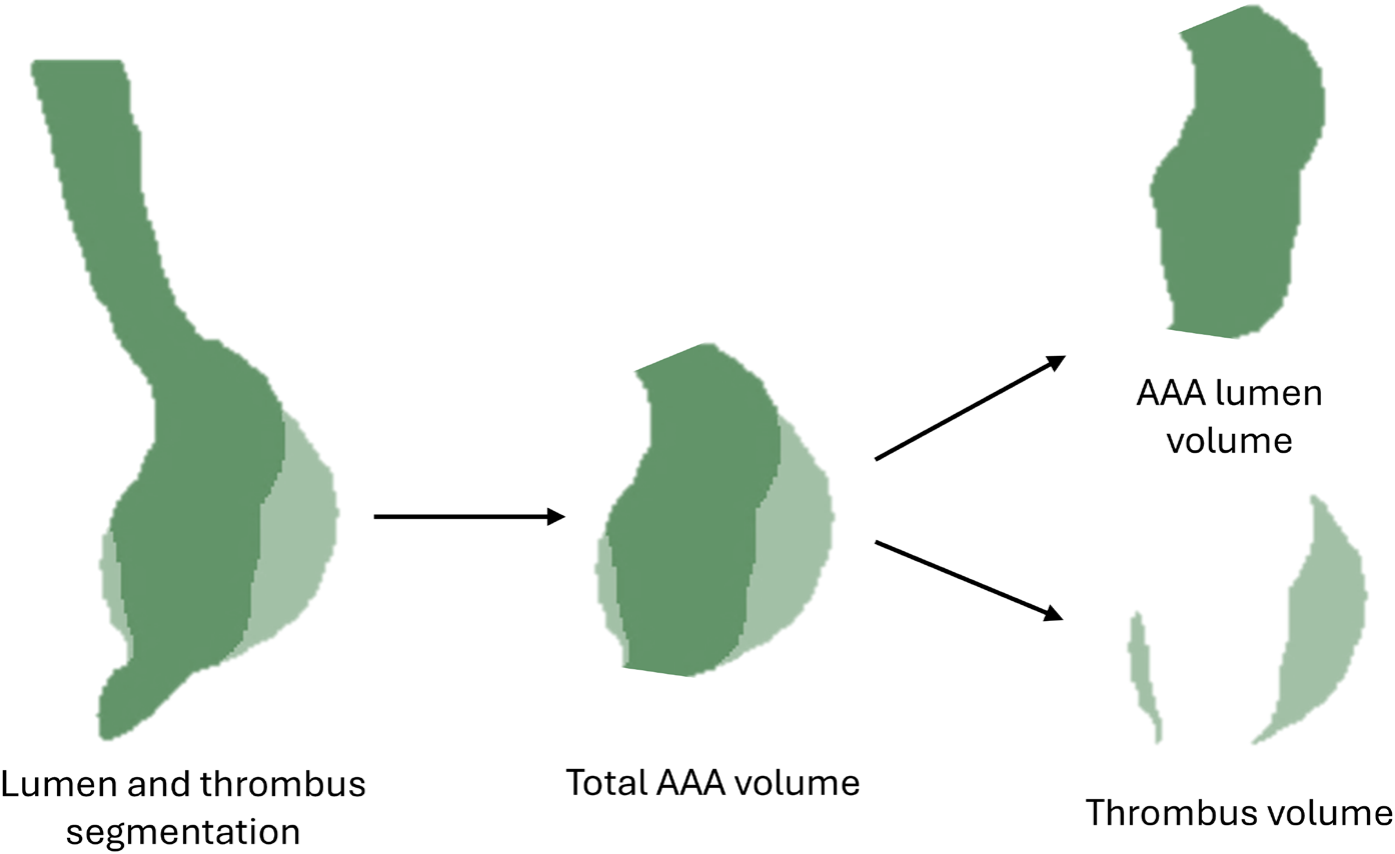
2D representation of the 3D volume post‐processing involving 3D volume calculations of total (thrombus + lumen) AAA volume, AAA lumen volume, and thrombus volume.

Based on the aneurysm extraction and the 3D cine bSSFP segmentation, the AAA lumen and thrombus volumes were calculated. Total AAA volume refers to the entire volume of the extracted aneurysm, including thrombus, whereas AAA lumen volume excludes the thrombus, as visualized in Figure 2. Patients were classified into two groups depending on the presence or absence of thrombus.

### 2.5 Visual Assessment of WSS Maps

In addition to quantitative analysis, a visual assessment of the WSS maps was performed to gain qualitative insight into spatial WSS patterns that may differ between baseline and follow‐up. While global metrics (minimum and maximum mean and peak WSS) describe values averaged over the entire aneurysm and regional analysis with sub‐regions reduces the extent of spatial averaging, localized WSS changes may still be obscured. Visual inspection of the WSS maps was therefore included to support interpretation of the quantitative findings and to better understand the data. Two observers (EA and ES) with respectively 4 and 15 years of experience with 4D flow MRI analysis performed the visual assessment of the WSS maps.

### 2.6 Statistical Analyses

Continuous parameters that followed a normal distribution are depicted as mean ± standard deviation (SD), while for non‐normally distributed data, median with interquartile range (IQR) was utilized. Normality of the collected data was evaluated through the Shapiro–Wilk test. The Wilcoxon signed‐rank test was applied to evaluate differences between measurements and patient subgroups. Specifically, this test was used to: compare WSS measurements, diameter measurements, AAA lumen volume, and thrombus volume between baseline and follow‐up; and to evaluate differences in WSS values between patient groups stratified by thrombus presence and location. The intraclass correlation coefficient (ICC) was calculated to assess inter‐observer reproducibility with a two‐way mixed effects model with absolute agreement. To determine statistically significant growth, the standard error of the measurement (SEM) was calculated based on the following formula: $\mathrm{SEM}=\mathrm{SD}\times \sqrt{1-\mathrm{ICC}}$. Aortic growth was deemed statistically significant if: AAA diameter measurement at follow‐up > (AAA diameter at baseline +2 × SEM) [16]. Spearman's rank correlation coefficients were calculated to assess correlations between the half‐year growth of the AAA, as defined by the radiologists, and the (change in) WSS values, AAA lumen volume, AAA total volume and thrombus volume. The four included WSS parameters: minimum mean, maximum mean, minimum peak, and maximum peak, reflect related aspects of the same underlying dataset. Given the simultaneous testing of multiple WSS parameters, Bonferroni correction was used to control the family‐wise error rate and statistical significance was set at a two‐tailed *p*‐value of 0.0125 (*p* = 0.05/4). Statistical analyses were performed with R software (R Core Team, version 1.4.1717, Vienna, Austria).

## 3 Results

### 3.1 Study Population

**FIGURE 3:**
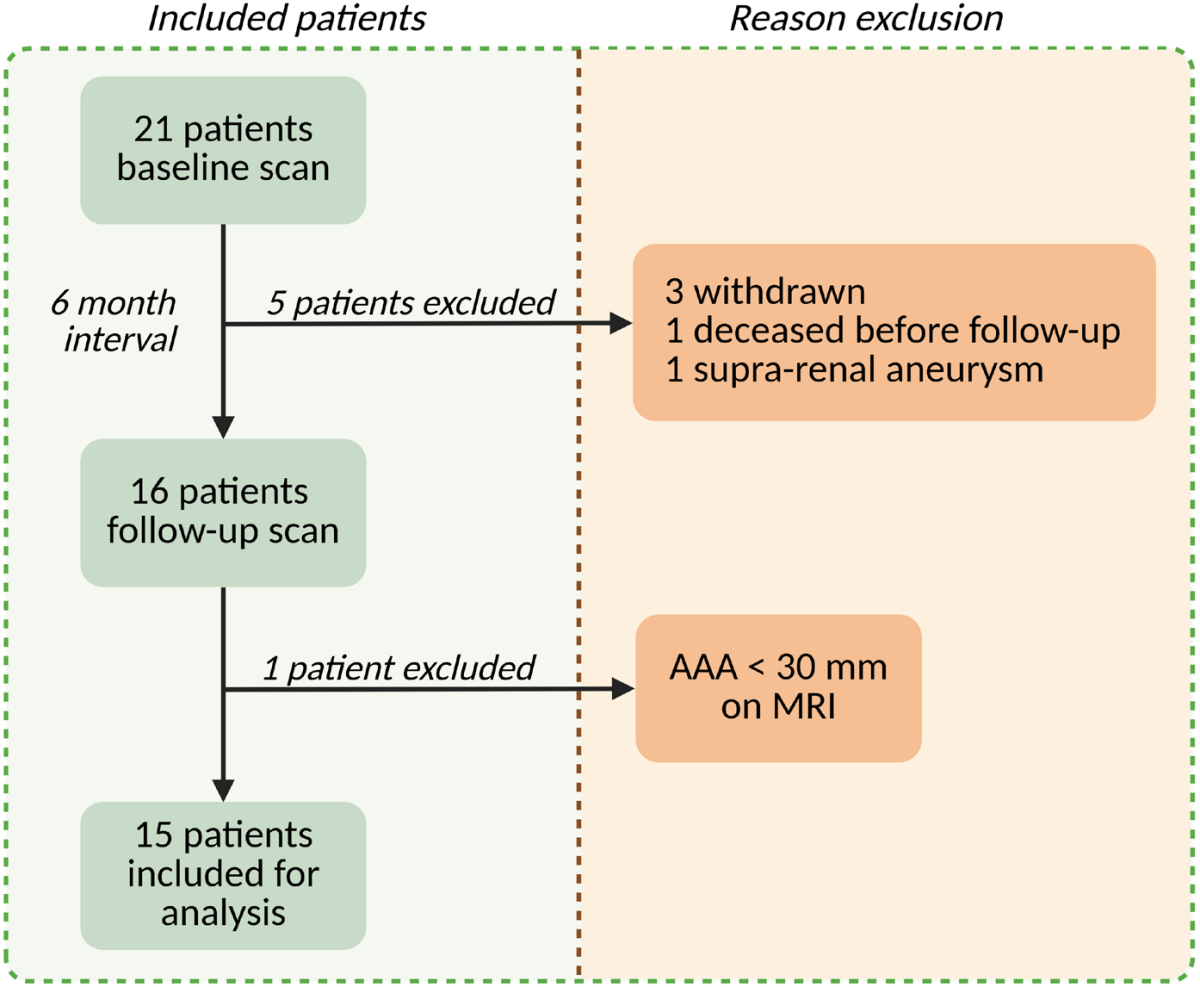
Flow chart illustrating patient inclusion and exclusion, with reasons for exclusion.

Twenty‐one AAA patients underwent a baseline MRI scan, of whom 16 also completed a follow‐up scan, see Figure 3. Based on diameter measurements, one patient was excluded because the AAA diameter was < 30 mm in diameter and < 1.5 times the proximal aorta diameter on MRI. Consequently, 15 patients (mean age 68.7 ± 8.9 years; one female) remained for analysis. The median time between the two scans was 6.41 (0.59) months. Baseline characteristics are shown in Table 1. Two patients underwent surgical AAA repair (one endovascular aortic repair and one open surgical repair) after participation in this study.

**TABLE 1:** Patient characteristics for all included patients at baseline. Some patients had multiple cardiovascular morbidities. Continuous variables are given as mean ± standard deviation or as median with IQR.

General features	
Age at inclusion (years)	68.7 ± 8.9
Gender (male)	14 (93.3%)
Weight (kg)	85.2 ± 14.9
Height (cm)	178.9 ± 8.9
Left–right AAA diameter (mm)	45.7 (13.3)
Diabetes	3 (20%)
Thrombus present	12 (80%)
Cardiovascular comorbidities	
Hypertension	4 (26.6%)
PAD	2 (13.3%)
Aorta valve stenosis	1 (6.7%)
Myocardial infract	1 (6.7%)
Iliac aneurysm	1 (6.7%)
Deep vein thrombosis	1 (6.7%)
Hypercholesterolemia	1 (6.7%)
Smoking status	
Current smoker	2 (13.3%)
Former smoker	10 (66.7%)
No smoker	3 (20.0%)
Elective surgery after study participation	
EVAR	1 (6.7%)
OSR	1 (6.7%)

Abbreviations: AAA = abdominal aortic aneurysm; EVAR = endovascular aorta repair; IQR = interquartile range; OSR = open surgical repair; PAD = peripheral artery disease.

### 3.2 Data Acquisition

4D flow MRI acquisition was successful in all patients. In four cases (three patients), the velocity encoding was increased to 75 cm/s to prevent aliasing. Due to time constraints, 4D flow MRI acquisition was terminated early in seven cases. In all cases, more than 70% of the target data acquisition was completed before early termination. Six out of the 30 segmentations were manually refined to ensure their quality for subsequent analysis. In one patient, suboptimal automated aneurysm extraction required manual adjustment to enable reliable WSS estimation.

### 3.3 Wall Shear Stress Assessment

Median WSS values are given for both baseline and follow‐up studies in Table 2. Median change in both mean and peak WSS between the two time‐points was minimal. A significant difference was observed in maximum peak WSS between baseline and follow‐up. All other investigated WSS values showed no significant differences between baseline and follow‐up (see Table 2 for corresponding *p*‐values). For all eight sub‐regions, the median WSS values for both baseline and follow‐up are given in Supporting Information Table S2. No significant changes were found between baseline and follow‐up WSS values in any of the sub‐regions (see Table S2 for corresponding *p*‐values).

**TABLE 2:** Median WSS values with IQR for baseline, follow‐up and change between the two time‐points.

	Baseline	Follow‐up	Change	Wilcoxon signed‐rank test
Mean WSS (Pa)				
Min	0.05 (0.01)	0.05 (0.02)	0.00 (0.01)	*p* = 0.208
Max	0.17 (0.09)	0.14 (0.15)	0.01 (0.02)	*p* = 0.055
Peak WSS (Pa)				
Min	0.10 (0.03)	0.10 (0.04)	0.01 (0.04)	*p* = 0.169
Max	0.32 (0.17)	0.37 (0.20)	0.05 (0.12)	***p*** **=** **0.008**

*Note*: Bold values indicate statistical significance.

Abbreviations: IQR = interquartile range; max = maximum; min = minimum; WSS = wall shear stress.

**FIGURE 4:**
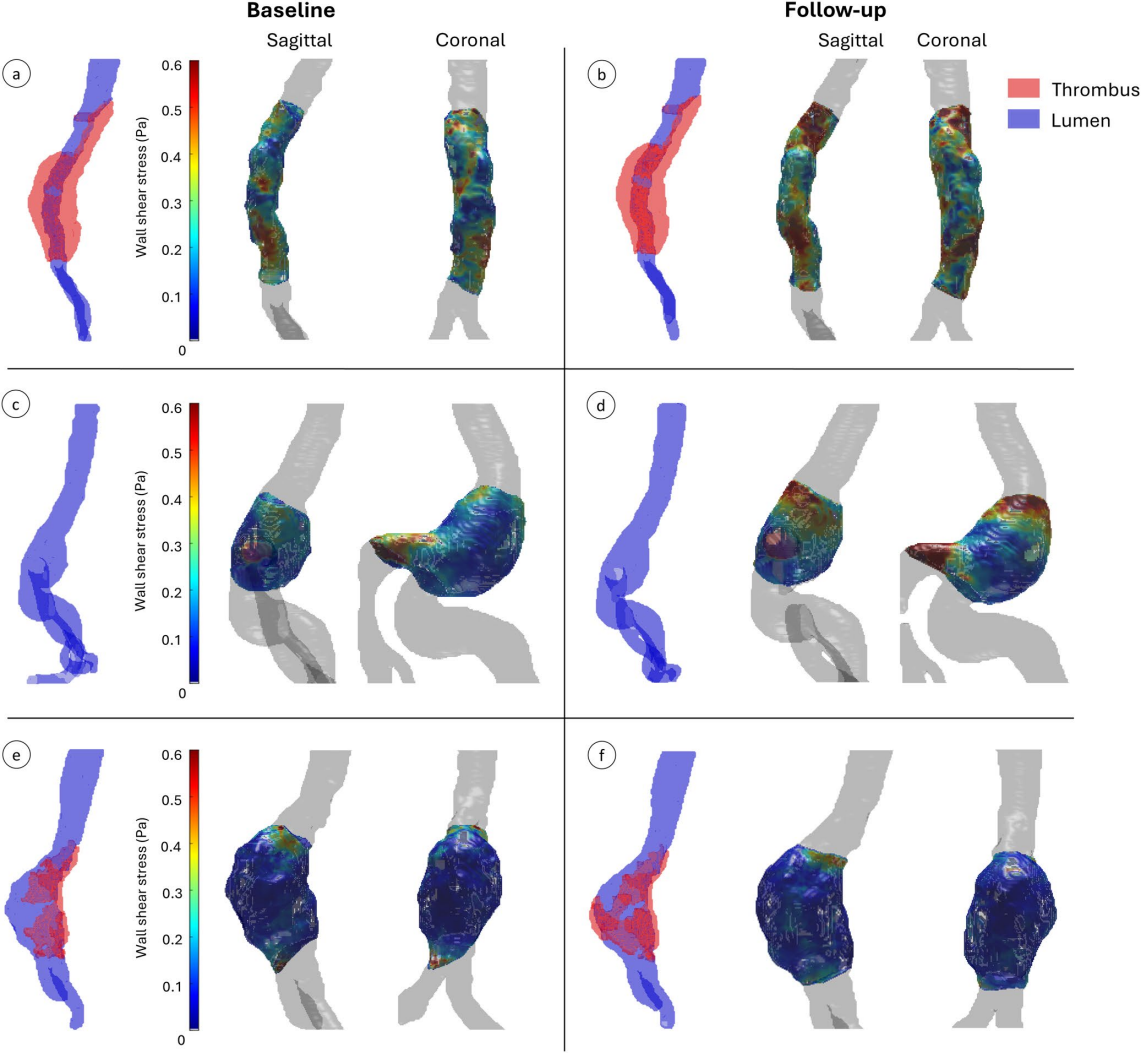
Three example cases are shown, each presented in a separate row. The first column (a, c, e) displays baseline data, while the second column (b, d, f) presents follow‐up data. In each panel (a–f), the first volume depicts the nnU‐Net segmentation of the lumen (blue) and, if present, the thrombus (red). The second and third volumes show WSS values within the extracted aneurysm at the peak systolic time frame, in the sagittal and coronal views, respectively. Case 1 (a–b) shows a patient with no growth but increased WSS values. Case 2 (c–d) shows a patient without thrombus and with a tortuous morphology who also showed no growth, but increased WSS values and case 3 (e–f) shows a patient with significant diameter growth where slight decreases in WSS values were observed.

Supporting Information Table S3 gives an overview of the changes in diameter, WSS values, AAA lumen volume, and thrombus volume over the 6‐month interval for each patient. Two‐dimensional (2D) representations of the WSS maps utilized for visual assessment are shown in Figure 4 depicting three example cases, one per row. For each case, WSS values at the peak systolic time frame are visualized in both the sagittal and coronal views, alongside the corresponding nnU‐Net based segmentation of lumen and, if present, thrombus. Regions of higher WSS values (red and yellow) correspond well between baseline and follow‐up. In the first case (Figure 4a,b; patient 9 in Table S3), all four WSS parameters increased at follow‐up based on the quantitative assessment, despite the absence of growth in AP‐ or LR‐directions and only negligible change in AAA lumen volume. Based on visual assessment, local increases in WSS were observed. The second case (Figure 4c,d; patient 10 in Table S3) involved a tortuous morphology without thrombus. Similarly, all WSS values increased based on the quantitative assessment, while diameter and lumen volume remained essentially unchanged. Particularly at the inflow and outflow of the AAA volume, increases in WSS were observed based on visual assessment. The final case (Figure 4e,f; patient 4 in Table S3) showed significant diameter growth, corresponding to an increase in AAA lumen volume accompanied with an increase in thrombus volume in the quantitative analysis. In this patient, most WSS values decreased slightly, except for peak minimum WSS. Additionally, WSS seemed stable in this patient based on visual assessment.

### 3.4 Diameter and Volume Assessment

The maximum diameters in the LR‐ and AP‐directions at baseline and follow‐up, as independently assessed by the three radiologists, are presented in Supporting Information Table S4. The inter‐observer reproducibility among the three radiologists was excellent (ICC = 0.97). At baseline, the median AAA diameter was 43.3 mm (IQR 12.8 mm) in AP‐direction and 45.7 mm (IQR 13.3) in LR‐direction. After 6 months, significant increases were observed, with a median AAA diameter of 44.3 mm (IQR 13.5 mm) in the AP‐direction and 48.0 mm (IQR 14.0 mm) in the LR‐direction. Based on the calculated SEM (1.77 mm), aortic growth was considered significant if it exceeded 3.54 mm. This was observed in only one patient (male, 67 years old, current smoker) with 4.0 mm growth in the AP‐direction. Grouping the data based on significant growth was therefore not possible. Furthermore, no significant changes were observed in AAA lumen volume (*p* = 0.15), AAA total volume (lumen + thrombus) (*p* = 0.45), and thrombus volumes (*p* = 0.47), between baseline and follow‐up. The percentage change relative to initial volume of thrombus and AAA lumen is depicted in the two last columns of Supporting Information Table S3. Median absolute percentage change for AAA lumen volume was 13.5% (IQR = 12.9%) and 14.3% (IQR = 19.8%) for thrombus volume.

### 3.5 Correlations With Temporal Changes in Wall Shear Stress

**FIGURE 5:**
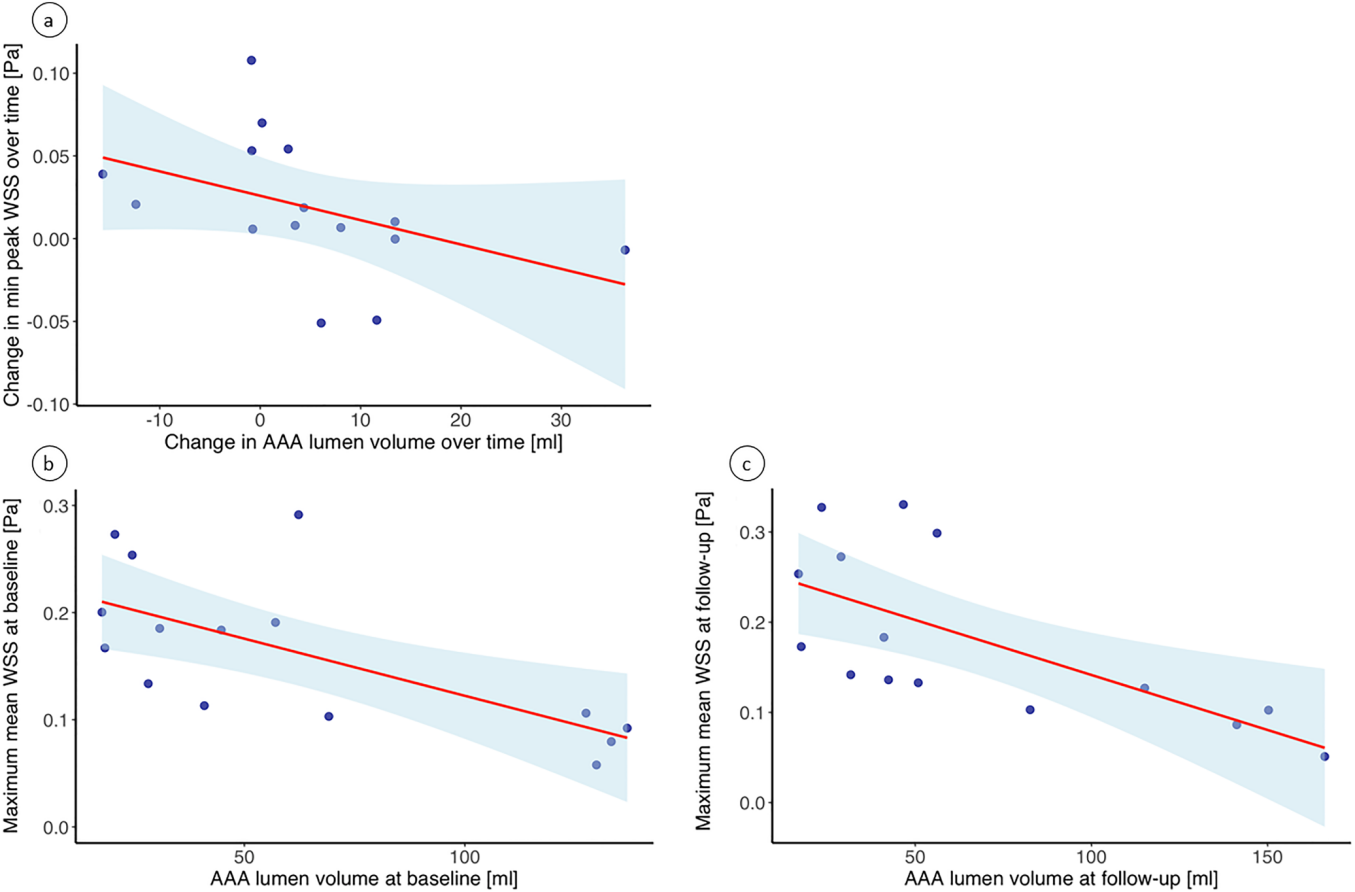
Linear regression of the observed inverse correlations. (a) Representation of the inverse correlation between the change in minimum peak WSS and AAA lumen volume over time. (b) Linear regression of the inverse correlation between maximum mean WSS and AAA lumen volume at baseline (c) and at follow‐up. The 95% CI is depicted in light blue.

The change in minimum peak WSS between baseline and follow‐up was significantly inversely correlated with the change in AAA lumen volume, encompassing both increases and decreases (*R* = −0.69), see Figure 5a. The values corresponding to the points in Figure 5a, are depicted in Supporting Information Table S3. After the Bonferroni correction, this was the only significant result concerning correlations between morphological parameters and changes in WSS, see Table 3. An inverse association between LR diameter change and mean WSS at both the minimum (*p* = 0.026, *R* = −0.57) and maximum time frames (*p* = 0.019, *R* = −0.60) was suggested. Thus, an increase in LR AAA diameter might be associated with a reduction in mean WSS values over 6 months. No associations were observed regarding diameter changes in AP‐direction and WSS [22, 23].

**TABLE 3:** Regression coefficients and *p*‐values for temporal changes in WSS over the time period and their association with maximum AAA diameter in LR‐ and AP‐direction, and AAA lumen, thrombus, and total AAA volume.

WSS	Maximum AAA diameter				AAA lumen volume		Thrombus volume		Total AAA volume	
	LR‐direction		AP‐direction							
	R	P	R	P	R	P	R	P	R	P
Min mean	−0.57	0.026	−0.45	0.091	−0.38	0.161	−0.14	0.651	−0.09	0.763
Max mean	−0.60	0.019	−0.44	0.103	−0.39	0.149	−0.10	0.766	−0.10	0.714
Min peak	−0.40	0.136	−0.36	0.186	**−0.69**	**0.005**	0.01	0.974	−0.34	0.211
Max peak	−0.41	0.128	−0.45	0.090	−0.61	0.019	0.01	0.991	−0.25	0.375

*Note*: Values in bold are statistically significant (*p* < 0.0125).

Abbreviations: AAA = abdominal aortic aneurysm; AP = anteroposterior; LR = left–right; Max = maximum; Min = minimum; WSS = wall shear stress.

### 3.6 Correlation Analysis of Wall Shear Stress Measured at Baseline and Follow‐Up

Both at baseline (*R* = −0.68) and follow‐up (*R* = −0.72), the maximum mean WSS values were significantly inversely correlated with AAA lumen volume, see Figure 5 b‐c. Moreover, an inverse trend was observed between maximum peak WSS and AAA lumen volume at follow‐up (*p* = 0.036, *R* = −0.55). Nevertheless, when evaluating total AAA volume and thrombus volume, no correlations were identified, see Table 4.

**TABLE 4:** Regression coefficients and *p*‐values for WSS values determined both at baseline and follow‐up separately and their association with AAA lumen, thrombus, and total AAA volume.

WSS	AAA lumen volume				Thrombus volume				Total AAA volume			
	Baseline		Follow‐up		Baseline		Follow‐up		Baseline		Follow‐up	
	R	P	R	P	R	P	R	P	R	P	R	P
Min mean	−0.29	0.294	−0.40	0.145	0.53	0.079	0.38	0.218	−0.01	0.974	−0.18	0.524
Max mean	**−0.69**	**0.007**	**−0.72**	**0.004**	0.43	0.161	0.30	0.342	−0.33	0.237	−0.46	0.086
Min peak	−0.28	0.314	−0.42	0.123	0.62	0.035	0.50	0.099	0.08	0.77	−0.10	0.714
Max peak	−0.52	0.051	−0.55	0.036	0.36	0.246	0.29	0.354	−0.33	0.232	−0.45	0.091

*Note*: Values in bold are statistically significant (*p* < 0.0125).

Abbreviations: AAA = abdominal aortic aneurysm; Max = maximum; Min = minimum; WSS = wall shear stress.

### 3.7 Intraluminal Thrombus Assessment

A significant difference was observed in the change in maximum peak WSS over the two time‐points between the thrombus (*N* = 12) and non‐thrombus (*N* = 3) groups. No significant differences were observed regarding minimum mean (*p* = 0.365), maximum mean (*p* = 0.365), and minimum peak (*p* = 0.070) WSS values between the thrombus and non‐thrombus groups. In 5 out of 12 patients with thrombus, the bulk was located anteriorly, as shown in the example provided in Figure 2.

## 4 Discussion

The purpose of this study was to assess longitudinal changes in 4D flow MRI‐derived WSS and to investigate associations with maximum AAA diameter and AAA lumen, thrombus, and total AAA volume over a 6‐month period. The automated post‐processing pipeline facilitated efficient and rapid post‐processing, necessitating manual adjustment in only one patient. A significant difference in maximum peak WSS values was observed between baseline and follow‐up. No significant WSS differences were detected in the regional analysis. AAA diameter in the AP‐ and LR‐direction increased significantly between baseline and follow‐up at the group level. In contrast, significant aneurysm growth at the patient level was detected in only one patient. This may be attributed to the short follow‐up period. Visual assessment of 4D flow–derived peak systolic WSS maps may be valuable for AAA surveillance, since localized variations in WSS can be identified that may be lost when relying on mean WSS values. An inverse correlation was observed between change in minimum peak WSS and change in AAA lumen volume over time. Additionally, a significant difference in WSS change over 6 months was observed between patients with and without thrombus.

Although maximum peak WSS differed significantly between baseline and follow‐up, no correlations were found between changes in maximum peak WSS and changes in morphological parameters. Furthermore, no differences were observed in other WSS values between baseline and follow‐up. This might be attributable to the limited sample size and inherent variability in WSS measurements, an insufficient follow‐up duration to capture true physiological alterations, limited sensitivity of the measurement technique, or a combination. In addition, changes in WSS may not directly or linearly correspond to short‐term changes in morphological parameters, since local hemodynamic effects may occur independently of global morphological changes.

The lack of significant aneurysm growth at the patient level precluded subdivision into groups based on progression. Consequently, it was not possible to investigate potential differences in WSS between patients with and without aneurysm progression. A longer follow‐up period may capture morphological progression of the AAA at patient level, which could in turn lead to changes in WSS. However, longitudinal 4D flow MRI studies investigating WSS in AAA are lacking. Nevertheless, several studies have examined WSS longitudinally in the thoracic aorta although with conflicting results. Korpela et al. [16] studied 30 patients with ascending aortic aneurysms and found that decreased WSS was associated with aneurysm growth over 1 year; however, the small number of patients with significant growth (6/30) warrants cautious interpretation of this result. In another study, involving children and adolescents with Marfan syndrome (mean follow‐up 3.5 years), regional systolic WSS remained largely stable, except for a significant reduction in the proximal inner descending aorta which was correlated with an enlarged diameter at that location. Third, a retrospective 4D flow MRI database study involving bicuspid aortic valve patients with ≥ 5 years of follow‐up found that flow‐induced elevated WSS was associated with higher rates of aortic dilatation [17, 19]. Although differing in population, aortic segment, and pathology, these findings suggest that the sensitivity of 4D flow MRI‐derived WSS is sufficient to detect changes over time and that extended longitudinal studies in AAA patients may provide more insight into (regional) changes in WSS.

In this study, regional changes in WSS were investigated by dividing the total AAA volume into eight sub‐regions. However, the irregular lumen geometry caused by thrombus complicated comparisons between patients, despite 4D flow MRI accurately reflecting patient‐specific WSS distributions. In previous studies involving thoracic aortic diseases, alternative regional approaches have been employed to facilitate inter‐patient comparisons such as anatomical planes [16, 32, 33] or incidence maps [35]. These strategies are less feasible in AAA due to anatomical variability, vessel tortuosity, and heterogenous thrombus morphology, which strongly influence hemodynamics and preclude reliable registration to a common reference geometry needed for incidence maps. Although inter‐patient comparisons using regional assessment methods are inherently complex in AAA, such regional analyses have the potential to yield important patient‐specific insights into aneurysm progression. Visual assessment, as explored in the present study, may have potential value for AAA surveillance with 4D flow MRI, since localized WSS variations could be detected on 4D flow–derived maps but were not always reflected in averaged WSS measures of the entire AAA or individual sub‐regions.

Because the follow‐up period was relatively short, most patients did not show significant aneurysm growth, resulting in a restricted range of diameter and volume changes. Consequently, the statistical power to detect correlations with morphological parameters was reduced and necessitates a nuanced interpretation. Inverse correlations were identified between WSS values and the AAA lumen volume at baseline, follow‐up, and over time. These inverse correlations between AAA lumen volume and WSS values were anticipated given the inherent relationship between flow velocity, of which WSS is a derivative, and lumen dimensions, such as diameter or volume. A trend toward association was suggested between the change in both minimum and maximum mean WSS and dilatation in diameter in LR‐direction during follow‐up. It should be noted that the measured LR‐direction diameters include not only the patent lumen but also thrombus in the majority of patients in our cohort. The suggested inverse correlations might therefore not solely be based on the relation between flow volume and WSS but were consistent with earlier research. Previously, significantly lower values of peak WSS were observed in AAA patients compared with age‐matched controls and healthy volunteers [13]. Furthermore, a reduction in WSS has been reported in dilated abdominal aortic wall segments compared with nondilated segments in the same patients [36]. The consistency of the results of the current study with previous studies suggests the robustness of both the data and the post‐processing pipeline. However, the absence of correlations between WSS values and maximum diameter in the AP‐direction and total AAA volume (both closely related to the clinically used maximum diameter, as US measurements are based on the AP‐diameter and total AAA volume reflects the lumen and thrombus dimensions) suggests that WSS values might be influenced by other factors which are independent of morphological parameters. This hypothesis is supported by the local changes in WSS observed during visual assessment in patients without morphological changes.

In this study, the 3D cine bSSFP sequence allowed evaluation of thrombus volume. Although the sample sizes were small (three patients without thrombus and twelve with thrombus), precluding definitive conclusions, a significant difference in maximum peak WSS change over the 6‐month follow‐up was observed between patients with and without thrombus, with all patients without thrombus showing an increase. While changes in WSS related to thrombus volume over time are sparsely reported, these findings align with earlier studies showing that regions of low WSS are associated with thrombus deposition [12, 22]. Hemodynamic changes likely contribute to thrombus initiation and growth, with platelets activated in regions of high WSS and deposited in low‐WSS regions [22]. Therefore, a regional approach and consideration of thrombus in hemodynamic analysis, as employed in this study, may offer additional insight.

## 5 Future Perspectives

Future validation of WSS as a biomarker for AAA progression requires sufficient longitudinal follow‐up. The median half‐year growth of 1 mm in both the AP‐ and LR‐directions observed in the current study was slightly higher than reported in previous studies [37, 38], likely due to the inclusion of three patients with baseline diameters exceeding the surgical threshold of 5.5 cm. Assuming a mean growth rate of 1.7 mm/year [37], and defining significant growth as 3.54 mm (as calculated in the present study), approximately half of the aneurysms would show significant growth after ≈2.1 years. A follow‐up of at least 2.5 years is therefore recommended to reliably detect aneurysm growth and enable stratification into growth and non‐growth groups. Alongside longitudinal follow‐up, adequate occurrence of key clinical endpoints, such as AAA rupture, AAA repair, or death, is essential. In a study evaluating a different MRI biomarker for AAA progression, 5% of 342 patients with baseline diameters > 40 mm experienced rupture during 2 years of follow‐up [39]. In a screening cohort of men with AAAs measuring 5.0–5.4 cm, the annual rupture rate was only 0.4% [40]. These small incidence numbers underline the need for a large sample size or imaging biobank to ultimately validate WSS as a biomarker for AAA progression.

## 6 Limitations

The follow‐up period was limited due to the project timeline. Given the 6‐month follow‐up, changes in WSS are expected to be small, making the measurements more susceptible to noise and variability and reducing confidence that observed differences reflect true physiological changes. In addition, the follow‐up period was likely too short to capture measurable AAA progression in most patients, further limiting the reliability of the findings. Studies with longer follow‐up are therefore needed to assess a possible relation between AAA progression and 4D flow MRI‐derived WSS. Second, the small sample size limits generalizability of the findings. Moreover, the role of thrombus location in regional WSS could not be reliably assessed, as the group with non‐anterior thrombus was small and highly heterogeneous in thrombus location and distribution. Another limitation was the use of a combined aortic and iliac artery centerline for maximum diameter measurements in the post‐processing pipeline. Near the iliac bifurcation, the centerline deviates from the central axis of the lumen, curving toward the iliac arteries. This deviation complicates diameter assessment in the aorto‐iliac transition zone and can produce artificially elevated values. In the automated pipeline, such false peaks were mitigated by removing unrealistic outliers [29], but in one patient, this correction failed, requiring manual aneurysm extraction. Furthermore, segmentations of the 3D cine bSSFP data were performed on the first cardiac time frame of the mean respiratory phase data, which does not represent the same phase of the cardiac cycle across all patients and scans, due to variations in heart rate. Movement of the abdominal aorta over the cardiac cycle might therefore cause discrepancies in aneurysm volume both among patients and between the two time‐points. These variations could affect the reliability of diameter and volume measurements based on the segmentations. Yet, visual inspection of time‐resolved segmentations indicated minimal motion of the abdominal aorta over the cardiac cycle, with only a few voxel‐level differences observed between diastole and systole. Lastly, substantial changes in AAA lumen and thrombus volumes were observed in some patients over the 6‐month interval. Relative percentage changes were particularly pronounced for thrombus volume, partly reflecting the small absolute thrombus volumes in certain patients, whereby minor numerical differences between time‐points result in disproportionately large relative changes. In addition, a portion of the observed volume differences between baseline and follow‐up is likely attributable to segmentation variability rather than true physiological change. Although the employed nnU‐Net demonstrated excellent performance for lumen segmentation and good performance for thrombus segmentation [29], segmentation‐related uncertainty may have influenced volume estimates to some extent. While manual segmentation is also subject to inherent variability, these limitations should be considered when interpreting the volumetric findings.

## 7 Conclusion

Automated WSS assessment from 4D flow MRI in AAA patients was technically feasible, necessitating manual adjustment in only one patient. A significant difference was observed in maximum AAA diameter (the clinical reference standard) and maximum peak WSS between baseline and follow‐up. However, no correlations were found between the change in WSS and maximum AAA diameter over time. But a significant inverse correlation was observed between the change in WSS and AAA lumen volume over the 6‐month period. Visual assessment of WSS maps might enhance 4D flow–informed AAA surveillance by capturing localized WSS changes that may be obscured when relying on averaged (sub‐regional) WSS values. A larger study with longer follow‐up would be required to differentiate between growth and non‐growth patients and allow for assessment of 4D flow MRI‐derived WSS as a predictor of AAA progression.

## Funding

This research is part of MARVY and supported by Health Holland (LSH‐TKI 25379).

## Supporting information


**Table S1:** Detailed acquisition parameters for the three imaging acquisitions used in the study.
**Table S2:** For all eight regions the median WSS values with IQR for baseline, follow‐up, and the change between time‐points.
**Table S3:** An overview of the changes in diameter (growth), WSS values, AAA lumen volume, and thrombus volume over the 6‐month interval for each patient. The difference in AAA lumen and thrombus volume is also given as a percentage relative to baseline volume.
**Table S4:** Exact and mean maximum diameter values in AP‐direction and LR‐direction as obtained by three radiologists in all subjects. Baseline scans are denoted by “1,” and follow‐up scans by “2.”
