# Supplementary material for: Longitudinal 4D Flow MRI‐Derived Wall Shear Stress in Patients With an Abdominal Aortic Aneurysm
Source: J Magn Reson Imaging. 2026 Feb 11;63(5):1380–91. doi: 10.1002/jmri.70248 (PMC13066534; doi:10.1002/jmri.70248)
Supplement: Supplementary file 1 — Table S1: Detailed acquisition parameters for the three imaging acquisitions used in the study. Table S2: For all eight regions the median WSS values with IQR for baseline, follow‐up, and the change between time‐points. Table S3: An overview of the changes in diameter (growth), WSS values, AAA lumen volume, and thrombus volume over the 6‐month interval for each patient. The difference in AAA lumen and thrombus volume is also given as a percentage relative to baseline volume. Table S4: Exact and mean maximum diameter values in AP‐direction and LR‐direction as obtained by three radiologists in all subjects. Baseline scans are denoted by “1,” and follow‐up scans by “2.” [file JMRI-63-1380-s001.docx]

## Supplementary Table S1

Table S1: Detailed acquisition parameters for the three imaging acquisitions used in the study.

|  | 4D flow MRI | 3D cine bSSFP | Dixon MRI |
| --- | --- | --- | --- |
| TR | 4.58 ms | 2.89 ms | 3,69 ms |
| TE | 2.78 ms | 1.44 ms | 1.19 ms |
| FA | 8° | 40° | 20 |
| Slice oversampling factor | 1 | 2.14 | - |
| Acquired spatial resolution | (1.6mm)^3^ | (1.6mm)^3^ | 1.6 x 1.7 x 3.5 mm^3^ |
| Reconstructed spatial resolution | (1.0mm)^3^ | (1.0mm)^3^ | 1 x 1 x 1.75 mm^3^ |
| Temporal resolution | ~ 40 ms | ~ 67 ms | - |

3D = three dimensional, 4D = four dimensional, bSSFP = balanced steady state free precession, FA = flip angle, TE = echo time and TR = repetition time.

## Supplementary Table S2

Table S2: For all eight regions the median WSS values with IQR for baseline, follow-up and the change between timepoints.

|  | Maximum mean | | | | Maximum peak | | | | Minimum mean | | | | Minimum peak | | | |
| --- | --- | --- | --- | --- | --- | --- | --- | --- | --- | --- | --- | --- | --- | --- | --- | --- |
|  | Baseline | Follow-up | Change | Wilcoxon | Baseline | Follow-up | Change | Wilcoxon | Baseline | Follow-up | Change | Wilcoxon | Baseline | Follow-up | Change | Wilcoxon |
| Upper | | | | | | | | | | | | | | | | |
| Right | 0.16 (0.16) | 0.23 (0.21) | 0.04 (0.06) | P = 0.57 | 0.30 (0.17) | 0.35 (0.33) | 0.03 (0.19) | P = 0.74 | 0.04 (0.01) | 0.04 (0.02) | 0.00 (0.02) | P = 0.74 | 0.07 (0.03) | 0.08 (0.03) | 0.00 (0.03) | P = 0.65 |
| Anterior | 0.17 (0.11) | 0.22 (0.20) | 0.03 (0.07) | P = 0.65 | 0.35 (0.17) | 0.41 (0.34) | 0.07 (0.19) | P = 0.39 | 0.05 (0.02) | 0.04 (0.02) | 0.00 (0.01) | P = 0.97 | 0.10 (0.04) | 0.09 (0.03) | 0.01 (0.02) | P = 0.84 |
| Left | 0.17 (0.09) | 0.17 (0.16) | 0.01 (0.08) | P = 0.68 | 0.30 (0.25) | 0.32 (0.26) | 0.08 (0.16) | P = 0.44 | 0.04 (0.01) | 0.04 (0.01) | 0.01 (0.01) | P = 0.54 | 0.07 (0.04) | 0.08 (0.03) | 0.02 (0.02) | P = 0.51 |
| Posterior | 0.13 (0.07) | 0.13 (0.09) | 0.02 (0.03) | P = 0.62 | 0.30 (0.16) | 0.37 (0.22) | 0.00 (0.17) | P = 0.87 | 0.04 (0.01) | 0.04 (0.01) | 0.00 (0.01) | P = 0.77 | 0.08 (0.03) | 0.08 (0.04) | 0.01 (0.02) | P = 0.97 |
| Lower | | | | | | | | | | | | | | | | |
| Right | 0.18 (0.19) | 0.23 (0.13) | 0.01 (0.09) | P = 0.65 | 0.45 (0.43) | 0.43 (0.42) | 0.07 (0.26) | P = 0.97 | 0.04 (0.02) | 0.04 (0.01) | 0.00 (0.02) | P = 1.00 | 0.09 (0.05) | 0.09 (0.04) | 0.00 (0.02) | P = 0.90 |
| Anterior | 0.16 (0.13) | 0.16 (0.12) | 0.03 (0.05) | P = 0.51 | 0.33 (0.20) | 0.34 (0.19) | 0.09 (0.14) | P = 0.49 | 0.04 (0.01) | 0.04 (0.01) | 0.00 (0.01) | P = 0.54 | 0.08 (0.03) | 0.09 (0.01) | 0.00 (0.02) | P = 0.84 |
| Left | 0.17 (0.11) | 0.15 (0.20) | 0.01 (0.04) | P = 0.97 | 0.30 (0.18) | 0.32 (0.28) | 0.09 (0.18) | P = 0.65 | 0.04 (0.01) | 0.04 (0.01) | 0.00 (0.01) | P = 0.74 | 0.08 (0.02) | 0.08 (0.04) | 0.00 (0.03) | P = 0.97 |
| Posterior | 0.12 (0.12) | 0.14 (0.10) | 0.02 (0.04) | P = 0.81 | 0.27 (0.19) | 0.34 (0.21) | 0.05 (0.10) | P = 0.46 | 0.04 (0.03) | 0.04 (0.02) | 0.00 (0.01) | P = 0.93 | 0.07 (0.03) | 0.09 (0.04) | 0.01 (0.02) | P = 0.35 |

IQR = interquartile range; max = maximum; min = minimum; WSS = wall shear stress

## Supplementary Table S3

Table S3: An overview of the changes in diameter (growth), WSS values, AAA lumen volume and thrombus volume over the six-month interval for each patient. The difference in AAA lumen and thrombus volume is also given as a percentage relative to baseline volume.

| Patient number | Growth (AP) [mm] | Growth (LR) [mm] | Diff. Mean min WSS [Pa] | Diff. Mean max WSS [Pa] | Diff. Peak min WSS [Pa] | Diff. Peak max WSS [Pa] | Diff. AAA lumen volume [ml] | Diff. AAA lumen volume [%] | Diff. thrombus volume [ml] | Diff. thrombus volume [%] |
| --- | --- | --- | --- | --- | --- | --- | --- | --- | --- | --- |
| 1 | 1.0 | 0.7 | 0.01 | 0.01 | 0.07 | 0.23 | 0.2 | 0.4 | - | - |
| 2 | 1.0 | 1.0 | 0.01 | 0.02 | 0.02 | 0.03 | -12.4 | -9,7 | -6.5 | -8,6 |
| 3 | 1.3 | 1.0 | 0.01 | 0.02 | 0.01 | 0.03 | 8.0 | 6,0 | 1.1 | 1,4 |
| 4 | 4.0 | 2.7 | -0.01 | -0.02 | 0.00 | -0.03 | 13.4 | 20,0 | 10.2 | 56,5 |
| 5 | 0.7 | 2.3 | 0.00 | 0.00 | -0.01 | -0.01 | 36.3 | 27,5 | -3.7 | -63,3 |
| 6 | 2.0 | 1.7 | 0.00 | 0.01 | 0.01 | 0.07 | 13.4 | -16,6 | - | - |
| 7 | 1.7 | 2.0 | -0.02 | -0.05 | -0.05 | -0.05 | 11.6 | 50,1 | -22.4 | -18,7 |
| 8 | 1.3 | 1.7 | 0.00 | 0.00 | 0.04 | 0.16 | -15.7 | -24,7 | -5.7 | -4,4 |
| 9 | 0.0 | 0.0 | 0.02 | 0.03 | 0.05 | 0.17 | 2.8 | 13,5 | 0.2 | 0,2 |
| 10 | 0.0 | 1.0 | 0.01 | 0.03 | 0.11 | 0.59 | -0.9 | -2,0 | - | - |
| 11 | 1.0 | 0.3 | 0.01 | 0.03 | 0.02 | 0.05 | 4.4 | 17,7 | 2.1 | 13,3 |
| 12 | 0.7 | 1.0 | 0.00 | -0.01 | 0.05 | 0.08 | -0.8 | -6,9 | 2.4 | 15,3 |
| 13 | 1.0 | 1.0 | 0.00 | 0.00 | -0.05 | -0.03 | 6.1 | 15,4 | 4.1 | 9,7 |
| 14 | 2.0 | 0.3 | 0.00 | 0.00 | 0.01 | 0.02 | 3.5 | 5,9 | -1.7 | -25,6 |
| 15 | -0.3 | -0.3 | 0.01 | 0.02 | 0.01 | 0.05 | -0.8 | -4,1 | -14.5 | -32,3 |

AP = anteroposterior; Diff. = difference; LR = left-right; max = maximum; min = minimum; WSS = wall shear stress

## Supplementary Table S4

Table S4: Exact and mean maximum diameter values in AP-direction and LR-direction as obtained by three radiologists in all subjects.

Baseline scans are denoted by “1,” and follow-up scans by “2.”

| Subject | Observer 1 | | | | Observer 2 | | | | Observer 3 | | | | Mean values | | | |
| --- | --- | --- | --- | --- | --- | --- | --- | --- | --- | --- | --- | --- | --- | --- | --- | --- |
|  | AP-1 [mm] | LR-1 [mm] | AP-2 [mm] | LR-2 [mm] | AP-1 [mm] | LR-1 [mm] | AP-2 [mm] | LR-2 [mm] | AP-1 [mm] | LR-1 [mm] | AP-2 [mm] | LR-2 [mm] | AP-1 [mm] | LR-1 [mm] | AP-2 [mm] | LR-2 [mm] |
| 1 | 40 | 41 | 40 | 41 | 39 | 39 | 41 | 40 | 40 | 40 | 41 | 41 | 39,7 | 40,0 | 40,7 | 40,7 |
| 2 | 54 | 54 | 54 | 55 | 52 | 53 | 54 | 54 | 54 | 53 | 55 | 54 | 53,3 | 53,3 | 54,3 | 54,3 |
| 3 | 64 | 67 | 65 | 67 | 66 | 62 | 69 | 63 | 69 | 62 | 69 | 64 | 66,3 | 63,7 | 67,7 | 64,7 |
| 4 | 47 | 48 | 49 | 50 | 47 | 46 | 53 | 50 | 49 | 50 | 53 | 52 | 47,7 | 48,0 | 51,7 | 50,7 |
| 5 | 44 | 43 | 45 | 44 | 52 | 50 | 52 | 54 | 45 | 44 | 46 | 46 | 47,0 | 45,7 | 47,7 | 48,0 |
| 6 | 58 | 56 | 58 | 57 | 56 | 56 | 60 | 58 | 56 | 55 | 58 | 57 | 56,7 | 55,7 | 58,7 | 57,3 |
| 7 | 57 | 57 | 58 | 59 | 57 | 58 | 61 | 59 | 59 | 57 | 59 | 60 | 57,7 | 57,3 | 59,3 | 59,3 |
| 8 | 52 | 54 | 53 | 54 | 53 | 53 | 54 | 55 | 51 | 52 | 53 | 55 | 52,0 | 53,0 | 53,3 | 54,7 |
| 9 | 42 | 40 | 42 | 40 | 41 | 41 | 41 | 41 | 40 | 40 | 40 | 40 | 41,0 | 40,3 | 41,0 | 40,3 |
| 10 | 37 | 38 | 37 | 39 | 38 | 40 | 38 | 42 | 41 | 41 | 41 | 41 | 38,7 | 39,7 | 38,7 | 40,7 |
| 11 | 38 | 37 | 39 | 38 | 41 | 40 | 41 | 40 | 38 | 39 | 40 | 39 | 39,0 | 38,7 | 40,0 | 39,0 |
| 12 | 36 | 36 | 37 | 37 | 36 | 38 | 37 | 39 | 39 | 39 | 39 | 40 | 37,0 | 37,7 | 37,7 | 38,7 |
| 13 | 25 | 27 | 21 | 22 | 28 | 25 | 28 | 25 | 26 | 26 | 26 | 26 | 43,3 | 53,0 | 44,3 | 54,0 |
| 14 | 41 | 52 | 43 | 53 | 44 | 53 | 44 | 54 | 45 | 54 | 46 | 55 | 40,3 | 40,3 | 42,3 | 40,7 |
| 15 | 41 | 40 | 42 | 40 | 39 | 40 | 43 | 40 | 41 | 41 | 42 | 42 | 40,0 | 39,3 | 39,7 | 39,0 |

AP = anteroposterior, LR = left-right
